# Heme activation by DNA: isoguanine pentaplexes, but not quadruplexes, bind heme and enhance its oxidative activity

**DOI:** 10.1093/nar/gkv266

**Published:** 2015-03-30

**Authors:** Nisreen Shumayrikh, Yu Chuan Huang, Dipankar Sen

**Affiliations:** 1 Department of Chemistry, Simon Fraser University, Burnaby, British Columbia V5A 1S6, Canada; 2 Department of Molecular Biology & Biochemistry, Simon Fraser University, Burnaby, British Columbia V5A 1S6, Canada

## Abstract

Guanine-rich, single-stranded, DNAs and RNAs are able to fold to form G-quadruplexes that are held together by guanine base quartets. G-quadruplexes are known to bind ferric heme [Fe(III)-protoporphyrin IX] and to strongly activate such bound hemes toward peroxidase (1-electron oxidation) as well as oxygenase/peroxygenase (2-electron oxidation) activities. However, much remains unknown about how such activation is effected. Herein, we investigated whether G-quadruplexes were strictly required for heme activation or whether related multi-stranded DNA/RNA structures such as isoguanine (iG) quadruplexes and pentaplexes could also bind and activate heme. We found that iG-pentaplexes did indeed bind and activate heme comparably to G-quadruplexes; however, iG-quadruplexes did neither. Earlier structural and computational studies had suggested that while the geometry of backbone-unconstrained iG-quintets templated by cations such as Na^+^ or NH_4_^+^ was planar, that of iG-quartets deviated from planarity. We hypothesize that the binding as well as activation of heme by DNA or RNA is strongly supported by the planarity of the nucleobase quartet or quintet that interacts directly with the heme.

## INTRODUCTION

G-quadruplexes make up a family of folded structures formed by single-stranded and guanine-rich DNAs and RNAs (1–5). Sequences known to fold to G-quadruplexes under physiological conditions can be of genomic origin or entirely artificial, including many aptamers obtained from *in vitro* selection (1–5) out of random-sequence DNA/RNA libraries. In a G-quadruplex guanines from one to four distinct DNA or RNA strands hydrogen bond together, in Hoogsteen fashion, to form guanine base quartets. G-quadruplexes are polymorphic, showing divers strand molecularities, orientations/topologies, as well as forming both inter- and intra-stranded folds. Wholly parallel, antiparallel, as well as combination strand orientations have been described for DNA G-quadruplexes; RNA G-quadruplexes typically adopt a parallel strand orientation. Of the physiological cations, K^+^, and to a lesser extent, Na^+^, specifically support the formation and stabilization of G-quadruplexes. They do so by complexing to multiple guanine keto functionalities, either within a given G-quartet or between successive G-quartets (6). Other cations known to support G-quartet formation include Rb^+^, NH_4_^+^, Tl^+^, Sr^2+^, Ba^2+^ and Pb^2+^. However, cations such as Mg^2+^ and Ca^2+^ do not specifically stabilize G-quartets, though they do stabilize G-quadruplexes via general electrostatic stabilization (6,7).

In recent years, compelling evidence has been gathered on the occurrence of G-quadruplexes *in vivo*, as well as of their likely role in a number of cellular processes and in key human diseases (2–5). Genomic elements implicated in forming G-quadruplexes *in vivo* include telomeres, oncogenic and other gene promoters, ribosomal genes, immunoglobulin switch regions and various repetitive satellite sequences (2–5). In particular, it has been shown that telomeric DNA sequences, when folded into G-quadruplexes *in vitro*, inhibit the enzymatic action of the telomere-extending enzyme, telomerase (8). *In vivo*, telomerase over-expression is a characteristic of the overwhelming majority of human cancers (8). Recently, massive expansion of a (G_4_C_2_)_n_ repeat within the human *C9orf72* gene has been found to be causal of certain neurodegenerative diseases, notably, familial amyotrophic lateral sclerosis and frontotemporal dementia (9,10). (G_4_C_2_)_n_ DNA, as well as the RNA transcribed from it, forms G-quadruplexes (11–13). It has been proposed that the RNA G-quadruplexes, localized as intracellular RNA foci in affected cells, behave in a toxic fashion, sequestering many important RNA binding proteins (13) as well as heme. (14)

G-quadruplexes have been found to be excellent targets for the binding of many different large- and small-molecule ligands (4,15). Our lab first demonstrated that the multifunctional metabolic cofactor, ferric heme [Fe(III)-protoporphyrin IX], binds tightly to various G-quadruplexes (16,17). The dissociation constant (*K_d_*) values for such heme–DNA and heme–RNA interactions can be as low as ∼10 nM (18). G-quadruplex–heme complexes show some striking features, including UV-vis spectra that strongly resemble those of hemoproteins such as metmyoglobin (17). Most notably, however, these complexes show significant DNA/RNA-enhanced oxidative activity (both 1-electron and 2-electron oxidation), with initial kinetics of substate oxidation (depending on the substrate) comparable to those reported for heme-utilizing protein enzymes, including peroxidases, peroxygenases and monooxygenases (19–22). Such oxidative activity by G-quadruplex–heme complexes requires oxidizing agents such as hydrogen peroxide or molecular oxygen in the presence of reducing agents such as NADH (23) or ascorbate (14). In addition to the above, a number of groups have carried out illuminating studies on the properties of G-quadruplex–heme complexes: these include study of the topology of G-quadruplexes that bind and activate heme, the oxidative inactivation of G-quadruplex-bound heme; heme-binding and activation by single G-quartets; and studies on metal ion usage and on the range of reducing substrates that are compatible with G-quadruplex–heme oxidative catalysis (24–33). Although discovery of the enhanced oxidative properties of G-quadruplex–heme complexes was initially made from *in vitro* selection (SELEX) experiments, the utility of such complexes has mostly been in the fields of bioanalytical chemistry and nanochemistry (21,34–36). Recently, however, we have proposed that RNA G-quadruplex–heme complexes may play an unfavorable role of sequestering and activating cellular heme in certain neurodegenerative diseases (14).

In spite of a large literature on G-quadruplex–heme complexes and their oxidative properties, many structural and mechanistic features of these systems remain incompletely characterized. The following are known to date: (i) hemes in these complexes are end-stacked upon rather than intercalated into G-quadruplexes (37); (ii) in the catalytically active complexes the heme iron is in a six-coordinate, high spin state (17,38); and (iii) there appears to be a requirement for an extended π-surface—such as the guanine quartets of a G-quadruplex provide. To date, neither duplexes nor folds other than G-quadruplexes formed by DNA and RNA have been shown to bind or activate heme. We therefore wished to investigate what specific features of G-quadruplexes (and their component G-quartets) were necessary for heme binding and activation.

**Figure 1.**
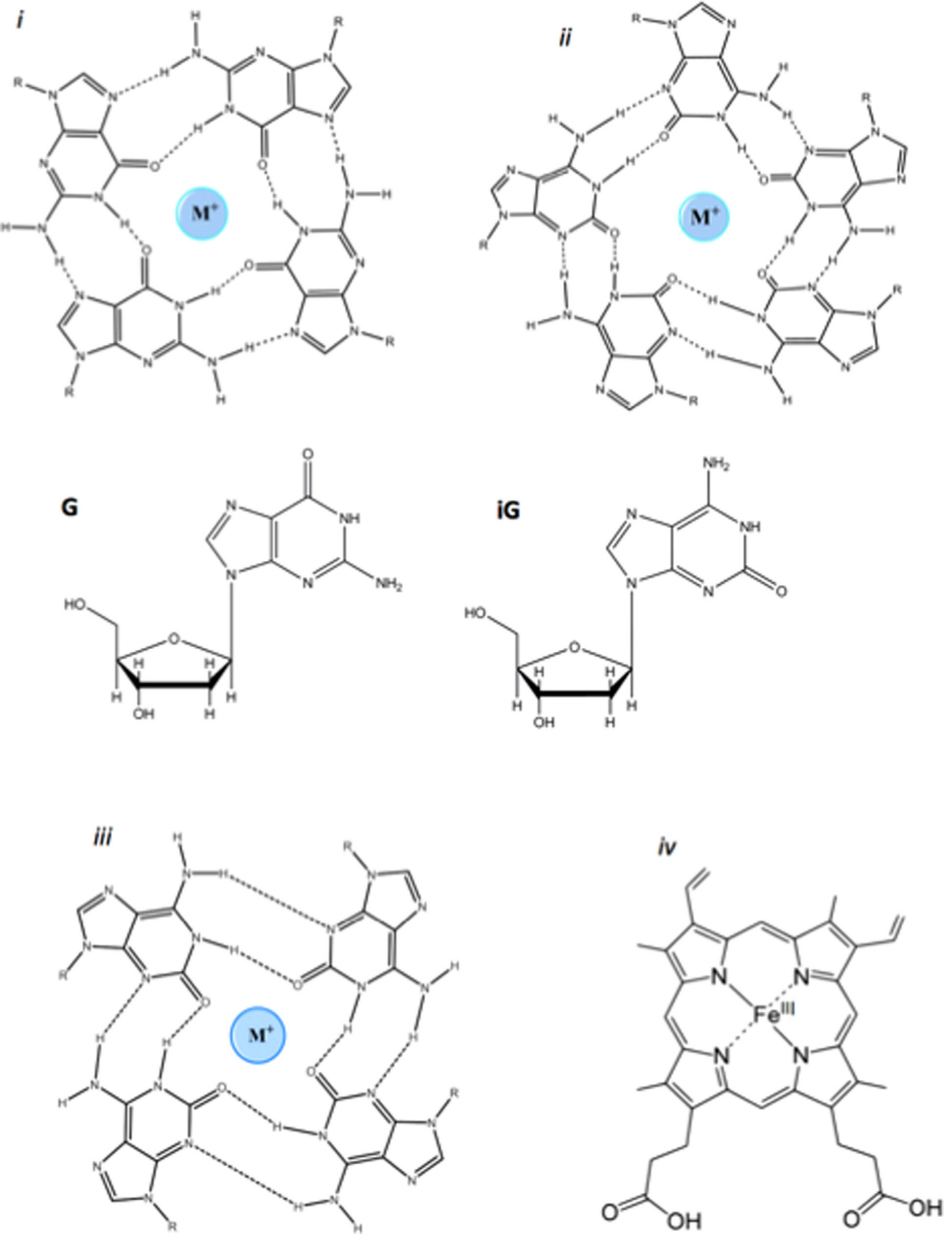
Chemical structures of 2′-deoxyguanosine (G), 2′-deoxyisoguanine (iG), guanine quartet (*i*); isoguanine quintet (*ii*); isoguanine quartet (*iii*); and Fe(III)-heme (*iv*).

Toward that end, we identified higher order structures formed by DNA containing the naturally occurring but non-genetic nucleobase, isoguanine (iG). A number of recent papers, by Chaput and Switzer (39) and by others (40–49), have probed the structure and properties of intermolecular multi-stranded complexes formed by isolated iG nucleosides, as well as by iG-containing single-stranded DNAs. It has been found that in the presence of specific cations (e.g. Na^+^, Rb^+^, Cs^+^, NH_4_^+^, Sr^2+^), the iG nucleosides form cation-templated iG-quartets or quintets. In the case of the DNA oligomers, parallel-stranded iG-pentaplexes are formed (and held together by iG quintets) in solutions of most of the above cations; however, in K^+^ solutions, especially at 0°C, iG-containing oligonucleotides form a parallel-stranded iG-quadruplex (held together by iG quartets) (39,50). Figure 1 shows the structures of (*i*) a G-quartet, (*ii*) an iG-quintet, (*iii*) an iG-quartet, and (*iv*) ferric heme.

We carried out a variety of spectroscopic and chemical experiments to, first, assemble and characterize iG-qudruplexes and pentaplexes, in order to investigate their heme-binding and activating properties, relative to standard G-quadruplexes.

## MATERIALS AND METHODS

### Materials

All DNA oligomers were purchased from the University Core DNA Services (University of Calgary) and size-purified by denaturing gel electrophoresis. Isoguanine phosphoramidite was purchased from Glen Research; Fe(III)heme (hemin) from Frontier Scientific; and γ-^32^P ATP was from PerkinElmer Life and from Analytical Sciences (33 μl, 185 Mbq; 6000 Ci/mmol). T4 polynucleotide kinase was from New England BioLabs Inc. (10 000 units/ml). All other chemicals and reagents were purchased from Sigma/Aldrich.

### Preparation of G-quadruplexes, iG-quintaplexes and iG-quadruplex

Relevant DNA oligonucleotides were denatured at 100°C for 3 min in TE (10-mM Tris, pH 7.5, 0.1-mM ethylenediaminetetraacetic acid (EDTA)) buffer, followed by cooling on ice. Eighty nanomoles of DNA were then incubated in 25-mM Tris-Cl, pH 7.5, supplemented with 500-mM potassium, sodium, ammonium or cesium chloride, at 25°C for 24 h. In experiments where multi-strand complexes were made by the mixing of two differently sized oligonucleotides, 5′-T_4_iG_4_T-3′ was mixed with 5′-T_8_iG_4_T-3′ (or 5′-T_4_G_4_T-3′ with 5′-T_8_G_4_T-3′) in 1:1 molar ratios. For the preparation of the iG-quadruplex from 5′-T_8_iG_4_T-3′, that oligonucleotide was incubated in 25-mM Tris-Cl, pH 7.5, supplemented with 500-mM potassium chloride, at 0°C, for 24 h.

### Circular dichroism spectroscopy

Circular dichroism (CD) experiments were carried out in a JASCO J-810 Spectropolarimeter at 25°C, using 0.05-cm path-length cuvettes. CD spectra were recorded both in the absence and presence of hemin. The final concentration of any given DNA multi-stranded complex was 10 μM, in 40-mM Tris-HCl, pH 8.0, 1% dimethylformamide (DMF), 0.05% Triton X-100. The final concentration of hemin was 0.5 μM. Spectra were recorded between 200 and 320 nm, and were averaged from three scans. CD measurements were also carried out at 0°C on the iG-quadruplex, assembled in a K^+^ buffer (see above). For experiments on the iG-quadruplex, in which NaCl and NH_4_Cl salts (to 20 and 35-mM final concentrations) were added post-assembly, incubation with the added salts was carried out for 24 h at 25°C. The CD spectra were then recorded for such solutions.

### Native gels

DNA complexes were prepared as described above and analyzed by electrophoresis through 20% acrylamide native gels, run at 4°C in 100-mM TBE (Tris/boric acid/EDTA) buffer, pH 8.0. For iG-containing DNA oligomers incubated with K^+^, the samples were analyzed in a gel, run at 4°C, in 100-mM TBE buffer, pH 8.0, supplemented with 5-mM KCl. The gels were exposed to phosphorous screens (Amersham Biosciences) for various times (3–5 h), at 4ºC, and the exposed screens were scanned in a Molecular Dynamics Typhoon 9410 Variable Mode Imager, to visualize radiolabeled bands. The gel images were then analyzed using either ImageQuant 5.2 (GE Healthcare) or ImageJ (NIH) software.

### Heme-binding assays

Heme-binding by all multi-stranded DNA complexes was monitored in a Varian Cary 300 bio UV-visible spectrophotometer, at 25 ± 1°C, using 10-mm quartz cuvette. A 5-mM hemin stock was prepared in DMF and stored in the dark at −20?C. Hemin was freshly diluted from the stock into the reaction buffer [40-mM Tris-HCl pH = 8, 1% DMF (v/v), 0.05% Triton X-100 (w/v)] containing multi-stranded DNA complexes at final concentrations of 0.2–20 μM, with either no further salt added (negative controls) or appropriate salt solutions (NaCl, NH_4_Cl, etc.) added to final concentrations of 20 mM. Spectra were collected from 200 to 800-nm wavelength.

### Calculation of binding constants

Absorbance data from titration experiments were used to construct saturation binding curves by plotting absorbance changes in the Soret band (404 nm) as a function of DNA concentration. Dissociation equilibrium constants (*K_d_*) were obtained by fitting the binding isotherm using nonlinear regression (OriginLab 9) with the following equation described by Wang *et al*. (51): [DNA]_0_ = *K_d_*(*A−A*_0_)/(*A*_∞_−*A*) + [*P*_0_](*A*−*A*_0_)/(*A*_∞_−*A*_0_), where [DNA]_0_ is the initial concentration of DNA, [*P*_0_] is the initial concentration of monomeric hemin (the concentration of hemin was calculated using ϵ_398_ = 80000 M^−1^ cm^−1^). *A*_∞_ indicates maximum hemin absorbance and *A*_0_ the initial absorbance in the absence of DNA.

### Peroxidase activity measurement

ABTS [2, 2′-azino-bis (3-ethylbenzothiazoline-6-sulfonic acid] was used as the oxidizable, chromogenic substrate. Peroxidation reaction was monitored by following the appearance of the oxidized ABTS^?+^ radical cation product, which absorbs light at 414 nm. For these assays, the final hemin concentration was 0.1 μM, in reaction buffer [40-mM Tris-HCl, pH 8.0, 20 mM of *X*Cl (where *X* is Na^+^, K^+^, Cs^+^ or NH_4_^+^), 1% DMF (v/v), 0.05% Triton X-100 (w/v)]. The ‘no salt’ reactions were monitored in reaction buffer itself, with no *X*Cl added. The final concentrations of ABTS and of the multi-stranded DNA complexes were 5 mM and 20 μM, respectively. Reactions were initiated with the addition of 1-mM H_2_O_2_ and were followed at 25°C. Peroxidase activity was measured also at 0°C for the iG-quadruplex and its controls.

## RESULTS

### CD characterization of multi-stranded DNA complexes

To generate G- and iG-mediated DNA strand-multimers, dT_8_G_4_T and, separately, dT_8_iG_4_T were first incubated in the buffered chloride solutions of various cations. The complexes formed by these strands, initially monitored by native gel electrophoresis, were then characterized using CD spectroscopy. CD is an excellent reporter of the strand orientations of such multiple-stranded DNA assemblies, although it does not provide the strand molecularities/stoichiometries of any such complexes.

**Figure 2.**
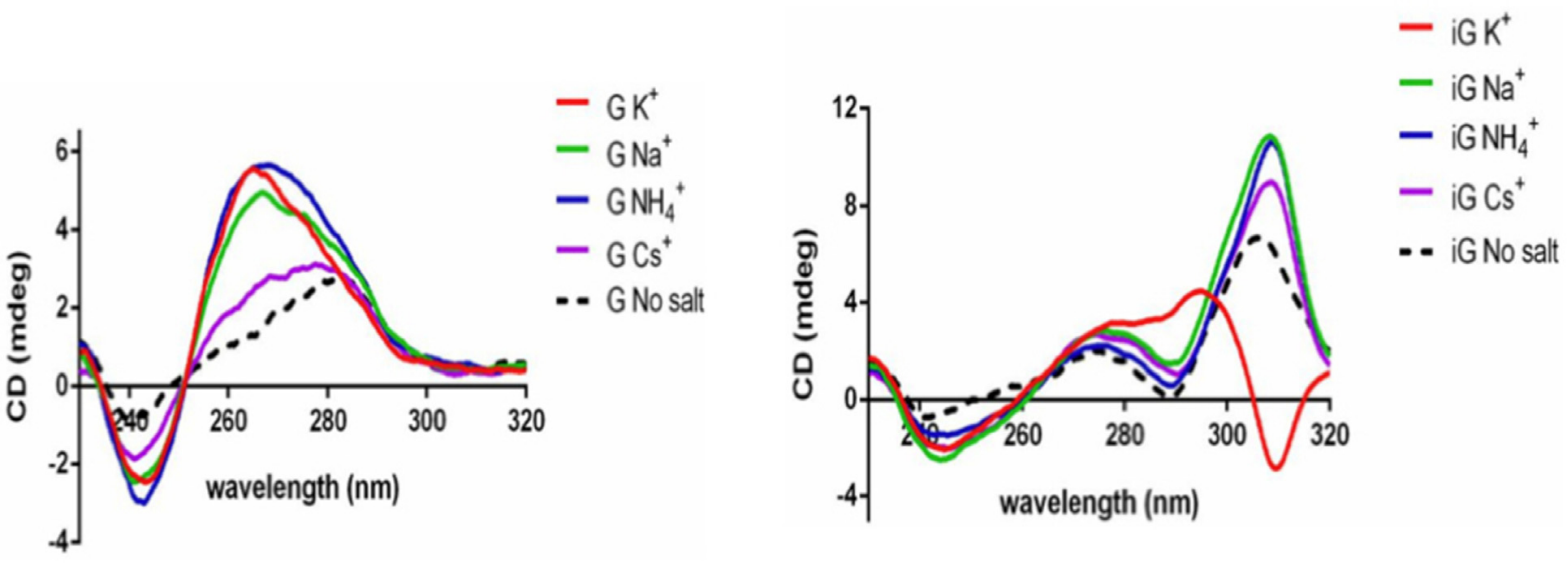
Circular dichroism (CD) spectra of the products of incubation of 5′-T_8_G_4_T (‘G’) and of 5′-T_8_iG_4_T (‘iG’), in buffered solutions containing, variously, 20 mM of NaCl, KCl, NH_4_Cl, CsCl or no added salt.

Figure 2 (left) shows the CD spectra of what are clearly G-quadruplexes formed by dT_8_G_4_T, in the presence of well-known G-quadruplex-promoting cations (Na^+^, K^+^, NH_4_^+^). All spectra show a strong positive peak at 260 nm and a negative peak at ∼245 nm; in all these cases, parallel-stranded G-quadruplexes are being formed. Incubations in Cs^+^, however, show only a modest amplitude enhancement over that of single-stranded T_8_G_4_T (without any added salt). Figure 2 (right) shows spectra for T_8_iG_4_T, incubated in the presence of the same set of cations, above. Here, two different kinds of spectra can be seen. In Na^+^, Cs^+^ and NH_4_^+^ solutions, maxima are seen at 275 nm (minor) and 310 nm (major), with a minimum at ∼245 nm. These spectra correspond to parallel-stranded iG pentaplexes, in agreement with earlier data by Pierce *et al*. (50). By contrast, in the K^+^ solution, the CD spectra are quite distinct, with a positive peak at ∼295 nm and a strong negative at ∼310 nm. These latter CD features have been proposed to correspond to an iG-quadruplex (50).

### Native gel analysis of the strand stoichiometries of iG pentaplexes and quadruplexes

To investigate the strand stoichiometries of complexes formed by T_8_G_4_T and T_8_iG_4_T in the different salt solutions, strand-mixing experiments were carried out. The number of strands in a multi-stranded DNA complex can be precisely determined by counting the total number of such complexes formed from mixtures containing two single-stranded DNAs (each with the identical G_4_ or iG_4_ motif but of different overall strand length) (7,39). Because strand multimers formed in such a mixing experiment would have different molecular weights (and, correspondingly, different electrophoretic mobilities), the formation of *n* multi-stranded complexes would indicate a strand molecularity of (*n*−1) for the complex.

**Figure 3.**
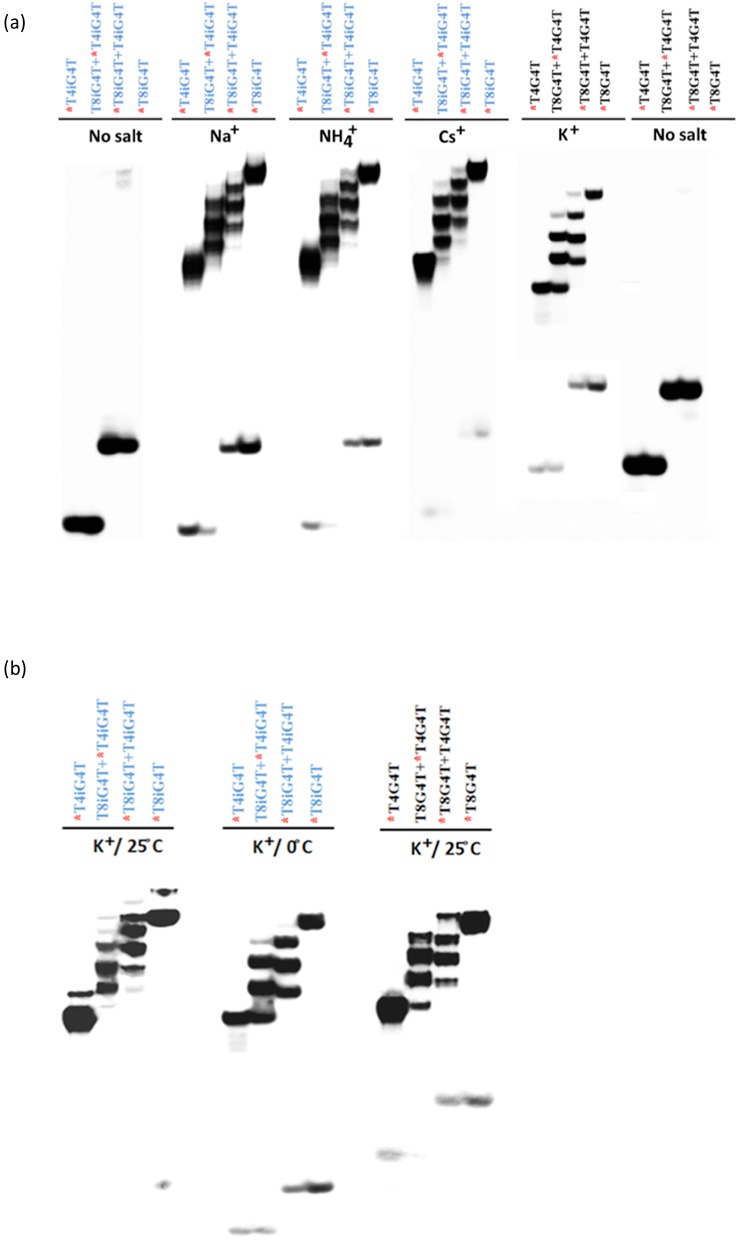
Native gel electrophoresis analysis of the multi-stranded products formed from incubation, with specific salt solutions, of 1:1 molar mixtures of 5′-T_4_G_4_T and 5′-T_8_G_4_T (labeled in black); or 5′-T_4_iG_4_T and 5′-T_8_iG_4_T (labeled in blue). Oligomers marked with a red asterisk are 5′-^32^P-labeled; those not so marked are not radiolabeled. (**a**) Incubations carried out at 25°C. (**b**) Incubations carried out at 25°C versus 0°C.

Figure 3a shows that co-incubation (at 25°C) of 1-mM T_4_iG_4_T with 1-mM T_8_iG_4_T, in solutions of 0.5 M Na^+^, Cs^+^ and NH_4_^+^, respectively, gives rise to a total of six closely spaced product bands of low electrophoretic mobility in each case. This indicates that these complexes are strand-pentaplexes. In contrast, incubation of the T_4_G_4_T/T_8_G_4_T with 0.5-M K^+^ gives rise to the expected five bands, corresponding to G-quadruplexes formed by these oligonucleotides.

The results of K^+^-promoted formation of strand-multimers, at different temperatures, from a mixture of T_4_iG_4_T and T_8_iG_4_T are shown in Figure 3b. Here, the temperature of incubation of (T_4_iG_4_T)_5_ and (T_8_iG_4_T)_5_ determines which product is obtained. In a 0°C incubation five product bands are seen, consistent with the formation of iG-quadruplexes; while the 25°C incubation gives rise to a more complex pattern of bands, indicating the formation of both iG-quadruplexes and iG-pentaplexes (39).

### Heme binding by iG pentaplexes and iG and G quadruplexes

**Figure 4.**
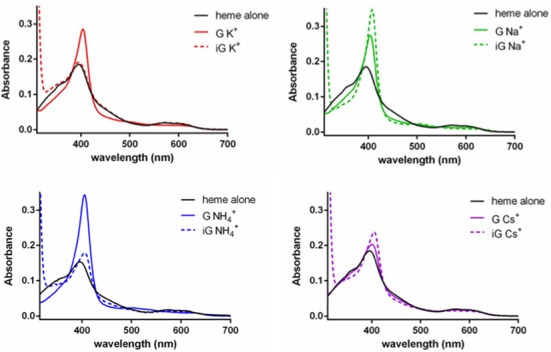
UV-vis spectra of 0.5-μM solutions of monomeric heme, following incubation with specific multi-stranded complexes formed by 5′-T_8_G_4_T (‘G’) and by 5′-T_8_iG_4_T (‘iG’) in buffered solutions containing, respectively, NaCl, KCl, NH_4_Cl and CsCl.

Do these various multi-stranded DNA complexes bind heme? In the case of G-quadruplexes, key UV-vis spectroscopic features indicative of heme-binding are well established (17); upon titration of a fixed concentration of monomeric heme with increasing concentrations of G-quadruplex, the following spectral features are seen: (i) an ∼2-fold hyperchromicity and modest red-shift (from ∼398 nm to 402–404 nm) of the heme's Soret absorption peak, and (b) characteristic changes in the heme's visible spectrum. Figure 4 shows the spectra of 0.5-μM hemin, dissolved in a buffer [40-mM Tris-HCl, pH 8.0, 1% dimethylformamide (v/v) and 0.05% Triton 100X (w/v)], supplemented with, variously, 20-mM final concentrations of KCl, NaCl, NH_4_Cl or CsCl. Figure 4 shows the spectra of monomeric heme, either in the absence of added DNA (‘heme alone’) or in the presence of 10 μM of (T_8_iG_4_T)_5_ [‘iG Na^+^’, ‘iG NH_4_^+^’ and ‘iG Cs^+^’]; (T_8_iG_4_T)_4_ [‘iG K^+^’]; and (T_8_G_4_T)_4_ [‘G K^+^’, ‘G Na^+^’, ‘G NH_4_^+^’ and ‘G Cs^+^’].

Inspection of the spectra of the Na^+^, NH_4_^+^ and Cs^+^ solutions shows that titration of ‘both’ the isoguanine pentaplex (T_8_iG_4_T)_5_ and the guanine quadruplex (T_8_G_4_T)_4_ to heme generates the classic signatures for heme–DNA complex formation (*vide infra*). In all cases, the red shift of the Soret band is observed, although the magnitude of Soret band hyperchromicity is somewhat variable. For instance, in NH_4_^+^ solution, G-quadruplex-binding elicits a larger Soret hyperchromicity relative to iG-pentaplex binding; however, that trend is reversed in Na^+^ solution. In Cs^+^ solution, titration of heme with either the G-quadruplex or iG-pentaplex shows relative modest changes in the UV-vis spectra; nevertheless, the changes seen are consistent with heme–DNA complex formation.

The most interesting results, however, are observed in K^+^ solutions. The G-quadruplex, (T_8_G_4_T)_4_, binds heme, as defined by the spectral changes. However, addition of the iG-quadruplex, (T_8_iG_4_T)_4_, to the heme (even at a quadruplex:heme ratio of 20:1) does not change the heme UV-vis spectrum at all. Such a lack of spectral change suggests that (T_8_iG_4_T)_4_ does not bind heme or does so with a very low affinity.

To determine binding affinities, titration of a fixed concentration (0.5 μM) of heme with various DNA multi-strand complexes was carried out. Supplementary Figure S1 shows UV-vis spectra of the heme titrated with 0–20-μM G-quadruplex, (T_8_G_4_T)_4_], and 0–20-μM iG-pentaplex, (T_8_iG_4_T)_5_], both in Na^+^ buffer. Dissociation constants (*K_d_*) values calculated were 3.22 ± 0.43 and 1.28 ± 0.05 μM, respectively. Supplementary Figure S2 shows that in K^+^ buffer, titration with 0–20-μM G-quadruplex [(T_8_G_4_T)_4_] yields a *K_d_* value of 7.89 ± 0.96 μM, whereas titration with even 70-μM iG-quadruplex [(T_8_iG_4_T)_4_] results in no discernable spectral change, consistent with a lack of heme binding to the iG-quadruplex.

To ensure that heme binding under these experimental conditions does not grossly change the structure of the DNA quadruplexes and pentaplexes, CD spectra of the multi-stranded structures, with and without added heme, were recorded. Supplementary Figure S3 shows that in no case is a substantial change in the CD spectrum seen following the addition of heme.

### Peroxidase activity of heme in the presence of excess iG pentaplex and quadruplex

**Figure 5.**
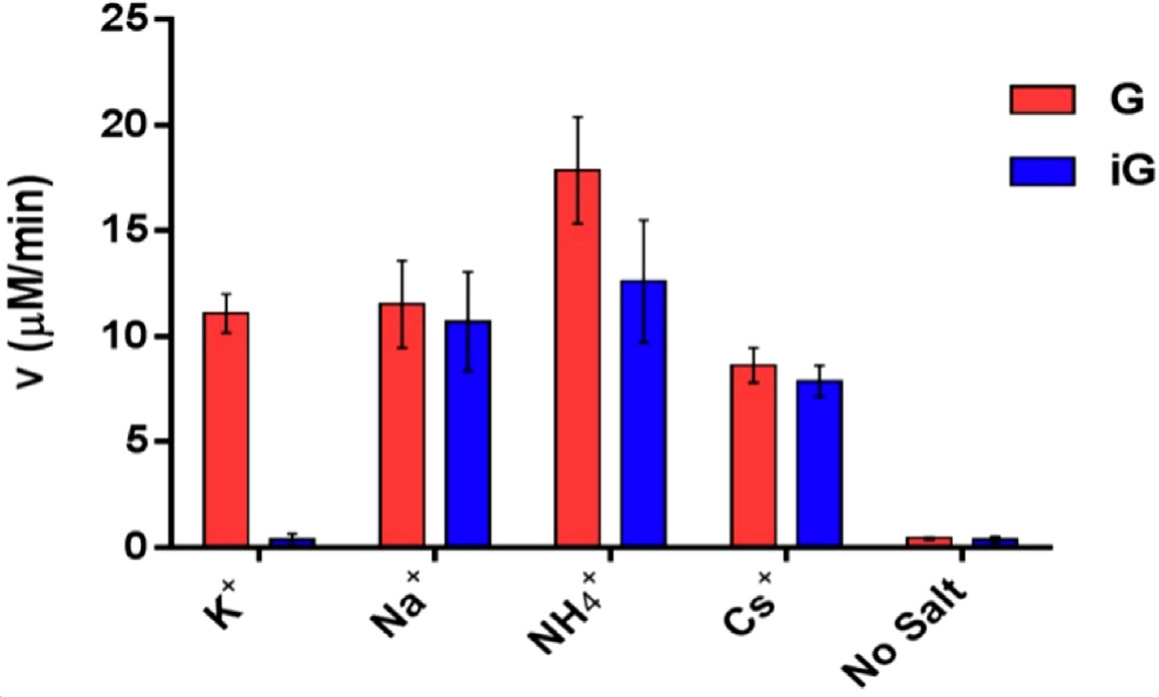
Peroxidase activity of 0.1-μM solutions of heme, in the presence of 20-μM multi-stranded product of either 5′-T_8_G_4_T (‘G’) or 5′-T_8_iG_4_T (‘iG’), formed in buffered solutions of, respectively, NaCl, KCl, NH_4_Cl and CsCl. Plotted are mean values, obtained from three independent experiments, of the reaction velocities of oxidation of the chromogenic substrate, ABTS [2,2′-azino-bis(3-ethylbenzothiazoline-6-sulphonic acid)], in the presence of 1-mM H_2_O_2_. Error bars indicate one standard deviation from the mean.

The peroxidase activity of proteinaceous heme enzymes, as well as of heme–DNA complexes, is conveniently monitored using hydrogen peroxide and a chromogenic substrate such as ABTS [2, 2′-azino-bis (3-ethylbenzothiazoline-6-sulfonic acid]. The raw data for ABTS oxidation by the various heme–DNA complexes are shown in Supplementary Figure S3. Figure 5 plots the observed rate constants (*k*_obs_) obtained from those kinetic data. Here, ‘G’ refers to data obtained from heme in solution with excess G-quadruplex, whereas ‘iG’ refers to corresponding data from excess iG-quadruplex and iG-pentaplex. It can be seen that in the Na^+^, Cs^+^ and NH_4_^+^ buffers, heme–G-quadruplex complexes (‘G’) show comparable oxidative behavior to heme–iG-pentaplex complexes (‘iG’). In the K^+^ buffer, the oxidative activity of the heme–G-quadruplex complex (‘G’) is comparable to those measured in Na^+^, NH_4_^+^ or Cs^+^ buffers; however, that of heme in the presence of excess iG-quadruplex shows only background activity. This is consistent with the observation, above, that the iG-quadruplex does not bind heme. Cumulatively, these data highlight an unexpected set of observations that (i) while both G-quadruplexes and iG-pentaplexes appear to manifest structural features capable of binding and activating heme, (ii) the iG-quadruplex, at least in a K^+^ buffer, does not present those features.

### The iG-quadruplex does not support peroxidase activity at different temperatures, or in the presence of Na^+^ or NH_4_^+^

**Figure 6.**
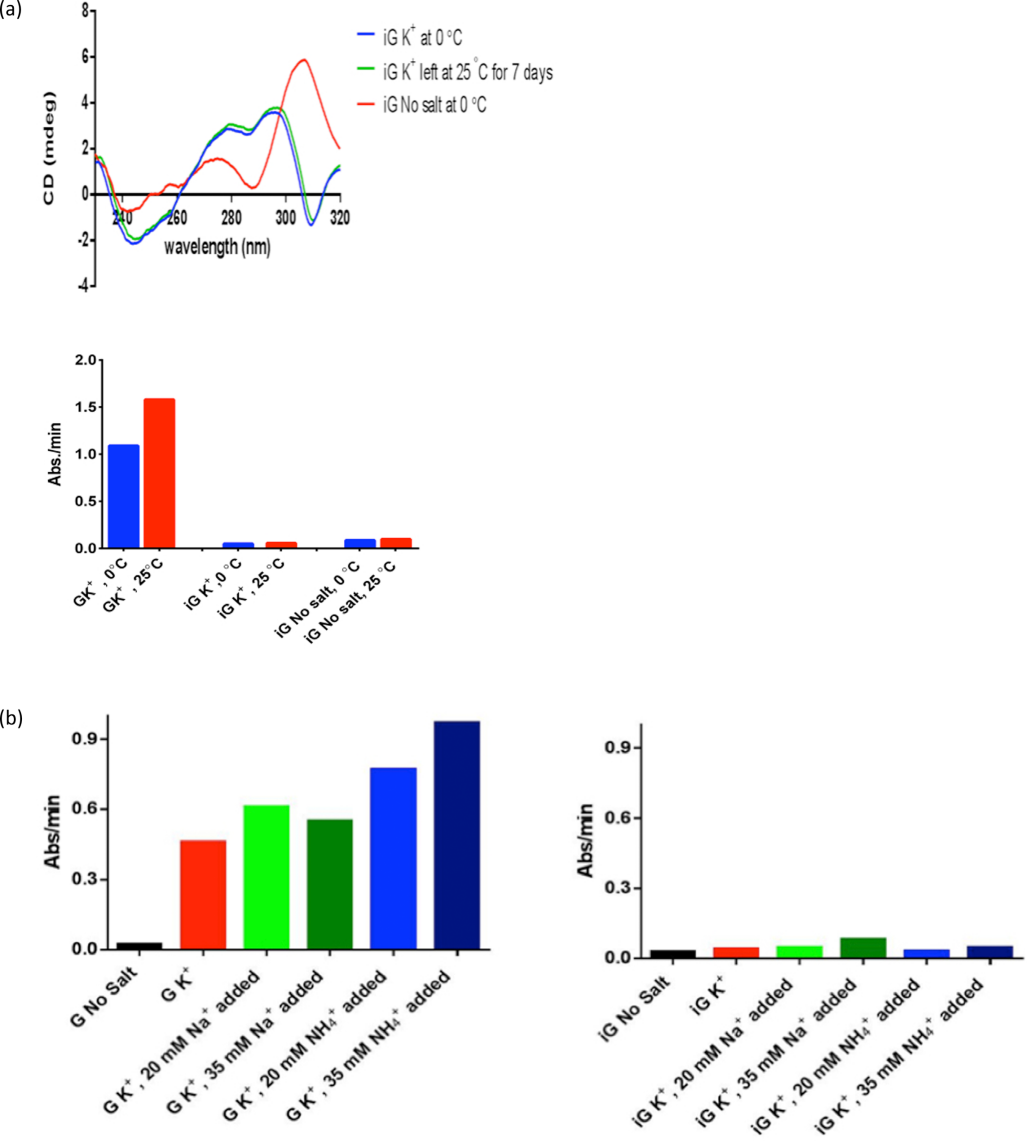
(**a**) Upper: Circular dichroism spectra of K^+^ buffer-generated iG-quadruplex at 0°C, as well as following incubation at 25°C for 7 days. The spectrum of the single-stranded 5′-T_8_iG_4_T (‘no salt’) at 0°C is shown for comparison. Lower: Peroxidase activity of heme in the presence of excess iG-quadruplex, at 0°C and 25°C, compared to that of heme in the presence of excess G-quadruplex, also at 0°C and 25°C. (**b**) Peroxidase activity (reported as absorbance/min) of 0.1-μM heme in the presence of 20 μM of the K^+^-generated G-quadruplex, (5′-T_8_G_4_T)_4_ (left), and of 20-μM K^+^-generated iG-quadruplex, (5′-T_8_iG_4_T)_4_ (right). Shown in red in either graph is the activity observed in K^+^ buffer alone. Bars shown in green and blue map activity observed in K^+^ buffers supplemented with Na^+^ and NH_4_^+^, respectively.

We wished to probe the effect of temperature on the structural stability of the K^+^ buffer-generated iG-quadruplex, as well as on the latter's ability to bind and activate heme at different temperatures. Thus, the K^+^-generated iG-quadruplex, formed at 0°C, was stored at 25°C for 7 days. Figure 6a, upper, shows that this storage at such a prolonged storage 25°C does not alter the CD spectrum of this multi-stranded structure. Therefore, the iG-quadruplex, once formed, does not readily disintegrate or reassemble as an iG-pentaplex, at least at 25°C. Figure 6a, lower, shows that solutions of 0.1-μM heme in the presence of either 10 μM of K^+^-generated iG-quadruplex or 40 μM of the single-stranded T_8_iG_4_T oligonucleotide (‘No salt’) show only baseline levels of peroxidase activity, both at 0°C and 25°C, whereas the K^+^-generated conventional G-quadruplex, under equivalent experimental conditions, shows peroxidase activity at both 0°C and 25°C.

With regard to the inability of the iG-quadruplex to bind or activate heme, we explored whether the K^+^ cation might play some inhibitory role in that inability. We had earlier shown that with G-quadruplex–heme complexes, cations such as Na^+^, K^+^ and NH_4_^+^ played a 2-fold role: first, the cations were necessary for the formation and stability of the G-quadruplex itself; however, NH_4_^+^/NH_3_ (as well as other nitrogenous base/conjugate acid systems, such as collidine/collidinium^+^) also substantially enhanced the peroxidase activity of G-quadruplex–heme complexes by means of general base catalysis (50). We therefore investigated whether the addition of NH_4_^+^ or Na^+^ ions to pre-existing K^+^-generated iG-quadruplex/heme solutions led to any enhancement of peroxidase activity. Figure 6b, left, plots the ABTS peroxidation *k*_obs_ values for heme–G-quadruplex complexes, in buffered 20-mM KCl solutions that have been supplemented with 20 or 35 mM of either NaCl or NH_4_Cl. It can be seen that while NaCl supplementations do not notably change the *k*_obs_ values, the addition of NH_4_Cl does lead to enhancements of the *k*_obs_ values, as expected. However, addition of either Na^+^ or NH_4_^+^ to the iG-quadruplex/heme solution does not improve on the background levels of peroxidase reaction (Figure 6b, right). To investigate the continuing structural integrity, or not, of the two DNA quadruplexes upon the addition of NH_4_^+^ or Na^+^ ions, CD spectroscopy was carried out. Supplementary Figure S3 shows CD spectra of both the G-quadruplex and iG-quadruplex, prior to as well as following addition of Na^+^ or NH_4_^+^. Changes in the CD spectra of either quadruplex are minimal upon addition of Na^+^. With NH_4_^+^, the amplitudes of the spectra change somewhat but the overall shape of each spectrum is maintained, suggesting that no gross rearrangement of structure occurs for either DNA quadruplex.

The above experiments contribute incremental evidence that the inability of the iG-quadruplex to enhance the peroxidase activity of heme lies in some structural property of the iG-quadruplex, which renders it a poor binding site for heme.

## DISCUSSION

Early studies on the formation of cation-tempated structures formed by iG nucleosides and iG-containing DNA strands were both structural and computational (40–43,45–49). Seela *et al*. (40,41) demonstrated the formation of tetra-stranded complexes by dT_4_iG_4_T_4_. DFT calculations by Meyer *et al*. (47,48) compared iG base quartets and quintets (lacking sugar-phosphate backbones) formed in the presence of Na^+^, K^+^, Rb^+^ ions and found that, in general, the quintets had relatively planar structures, whereas the quartets deviated from planarity. Even so, G-quintets templated by K^+^and Rb^+^ were calculated to be planar; however, those templated by Cs^+^ were not expected to be planar (48). Brodbelt *et al*. (49) combined ESI-mass and CD spectroscopy experiments with *ab initio* calculations to further examine the influence of the annealing cation, and reported that while G-quadruplex formation was strongly dependent on the identity of that cation, iG-pentaplexes were templated by a variety of cations (with the notable exception of K^+^, which favored the formation of iG-quartets from a combination of kinetic and thermodynamic factors) (39,49). The only experimentally determined high-resolution structure reported to date is from a nuclear magnetic resonance study, by Feigon *et al*., of a DNA iG-pentaplex formed by 5′-TiG_4_T (44). However, even in this Cs^+^-templated structure, the 5′-most of the four consecutive G-quintets has an almost planar structure, although the remaining three iG-quintets do indeed deviate significantly from non-planarity, as predicted (44).

Based on the above suggestive, albeit not conclusive, studies on the relative planarity of iG-quintets and iG-quartets, we hypothesize that our own observed lack of heme binding by the iG-quadruplex (as well as satisfactory heme binding by iG-pentaplexes) relates to the relative planarity of iG quintets within Na^+^ and NH_4_^+^-templated DNA iG-pentaplexes, and to deviation from such planarity of iG-quartets within K^+^-templated DNA iG-quadruplexes. Further high-resolution structural studies, especially on the iG-quadruplex, will clearly be required to throw light on this hypothesis.

In this study, we have shown unequivocally that the G-quartet is not the only nucleic acid ‘surface’ suitable for the dual property of heme binding and activation. The iG-quintet appears to function just as well, promising much for the potential future use of iG-pentaplexes in bioanalytical chemistry. For such a putative utilization, binding or catalytic event-led release of G-rich single-stranded DNAs would lead to their folding to iG-pentaplexes, to the binding and activation of heme, ultimately to generate luminescence, fluorescence or colorimetry-based output signals. An important question in this regard would be the relative chemical stability of iG-pentaplex DNA, compared to G-qudruplex DNA, in the presence of reactive oxygen species generated by DNA-activated heme and oxidants such as H_2_O_2_. An important experimental priority will be to determine, precisely, the relative chemical stabilities of the distinct DNA multi-strand structures. A higher durability of iG-pentaplexes under oxidative conditions would surely encourage their use over G-quadruplexes in practical applications.

An important question regarding the mechanism by which heme is activated by DNA, that remains to be fully elucidated is the following: is a large and planar surface, capable of π-stacking, sufficient to (a) bind and also (b) activate heme? In this study, heme binding and activation properties have been found to be tightly linked, inasmuch as activation necessitates binding. But are the two necessarily linked? It is conceivable that there exist DNA/RNA folded motifs that are capable of binding heme without activating it. We hypothesize that the ‘hole’ at the center of both G-quartets and iG-quintets is an important structural feature for the activation, though not necessarily for the binding of heme. We had earlier reported, on the basis of UV-vis (17) and EPR (38) data, that in a G-quadruplex bound heme (at physiological pH) the iron moiety was a six-coordinate, high-spin species. Such a 6-fold coordination of the iron necessarily implies the presence of an axial ligand in the direction of the G-quartet/iG-quintet upon which the heme is stacked. We propose that the presence of this yet unidentified axial ligand requires the central hole provided by G-quartets and iG-quintents, but not necessarily by other, extended base-paired structures in DNA and RNA that lack this structural feature. Experiments are currently in progress to test this hypothesis.

## SUPPLEMENTARY DATA

Supplementary Data are available at NAR Online.

SUPPLEMENTARY DATA
