# Supplementary material for: Heme activation by DNA: isoguanine pentaplexes, but not quadruplexes, bind heme and enhance its oxidative activity
Source: Nucleic Acids Res. 2015 Mar 30;43(8):4191–201. doi: 10.1093/nar/gkv266 (PMC4417173; doi:10.1093/nar/gkv266)
Supplement: SUPPLEMENTARY DATA [file supp_43_8_4191__index.html]

Heme activation by DNA: isoguanine pentaplexes, but not quadruplexes, bind heme and enhance its oxidative activity — SUPPLEMENTARY DATA 

# Heme activation by DNA: isoguanine pentaplexes, but not quadruplexes, bind heme and enhance its oxidative activity

## SUPPLEMENTARY DATA

**Files in this Data Supplement:**

- SUPPLEMENTARY DATA
