# Supplementary material for: Heme activation by DNA: isoguanine pentaplexes, but not quadruplexes, bind heme and enhance its oxidative activity
Source: Nucleic Acids Res. 2015 Mar 30;43(8):4191–201. doi: 10.1093/nar/gkv266 (PMC4417173; doi:10.1093/nar/gkv266)
Supplement: SUPPLEMENTARY DATA [file supp_gkv266_nar-00438-f-2015-File010.pdf]

Heme-Activation by DNA: Isoguanine Pentaplexes, but Not Quadruplexes, Bind Heme and Enhance its Oxidative Activity.

Nisreen Shumayrikh, Yu-Chuan Huang, and Dipankar Sen*

SUPPORTING INFORMATION

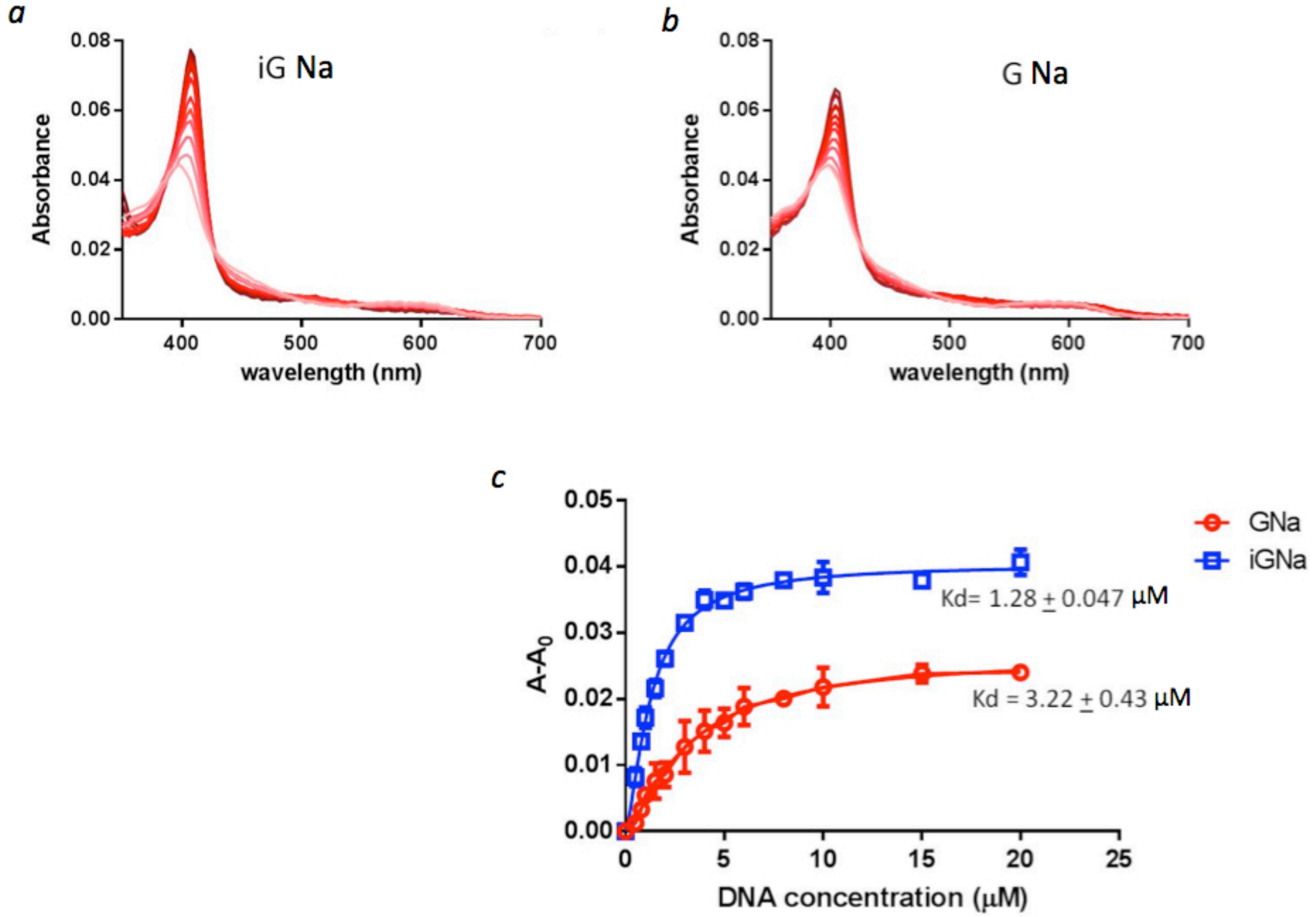

Figure S1: UV-vis spectra of 0.5 μM heme titrated with 0-20 μM multi-stranded DNA structures in a Na^+-buffer solution (40 mM Tris-HCl, pH 8.0, 20 mM NaCl, 1% DMF, 0.05% Triton X-100), at 25 °C. Titrations were carried out with ***a***: the iG-pentaplex, $(T_8iG_4T)_5$; and, ***b***: the G-quadruplex, $(T_8G_4T)_4$. ***c***: Plots of A-A_0 at 404 nm plotted against [multi-stranded DNA], to generate binding isotherms, and dissociation equilibrium constants (K_d) derived from them.

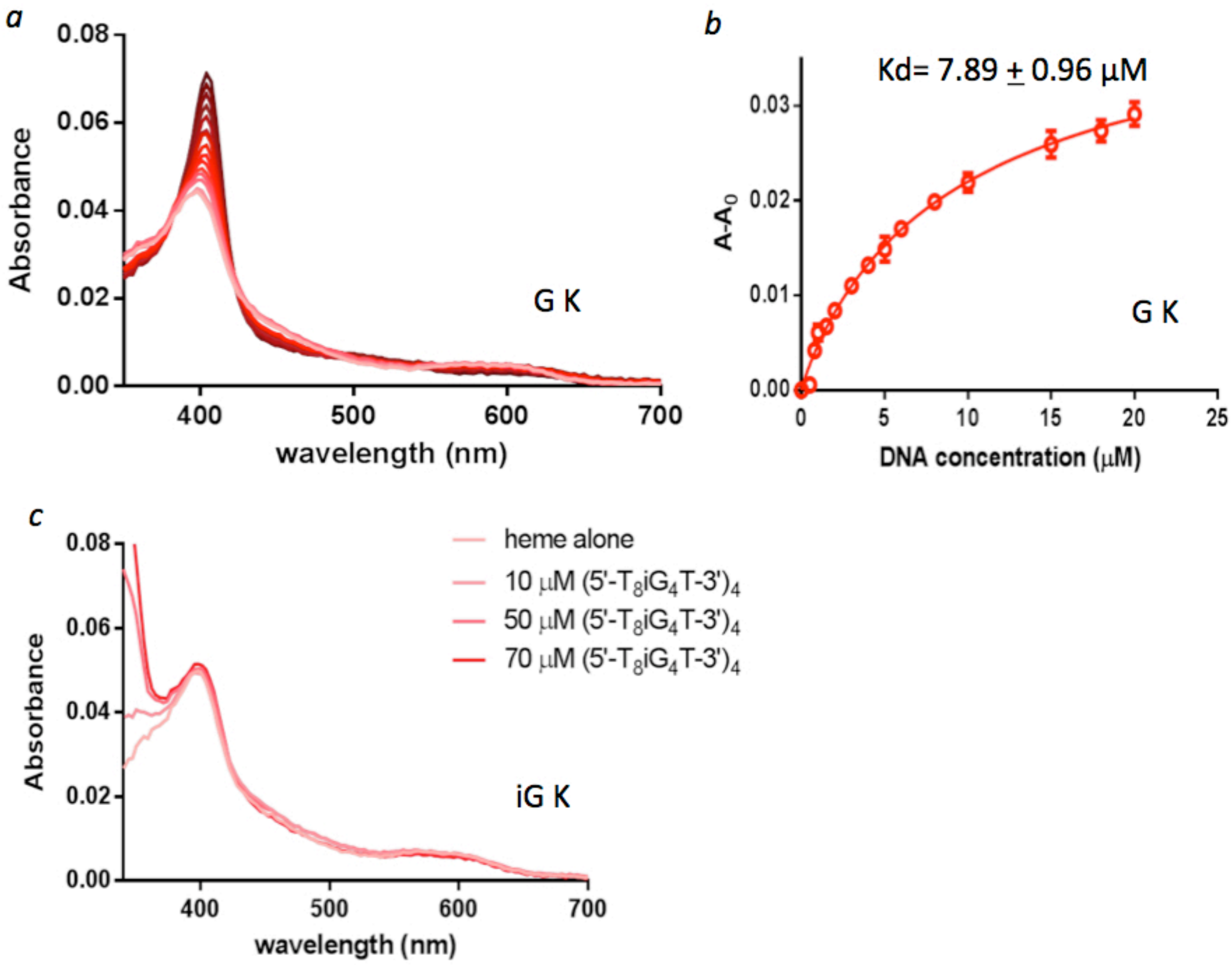

Figure S2: UV-vis spectra of 0.5 μM heme titrated with multi-stranded DNA structures in a K^+-buffer solution (40 mM Tris-HCl, pH 8.0, 20 mM KCl, 1% DMF, 0.05% Triton X-100), at 25 °C. ***a***: Titrations were carried out with the G-quadruplex, $(T_8G_4T)_4$, 0-20 μM. ***b***: Plot of $A\text{-}A_0$ at 404 nm plotted against $[(T_8G_4T)_4]$, to generate a binding isotherm, and the dissociation equilibrium constants (K_d) calculated from it. ***c***: Plot of titration of 0.5 μM heme with 10-70 μM iG-quadruplex, $(T_8iG_4T)_4$.

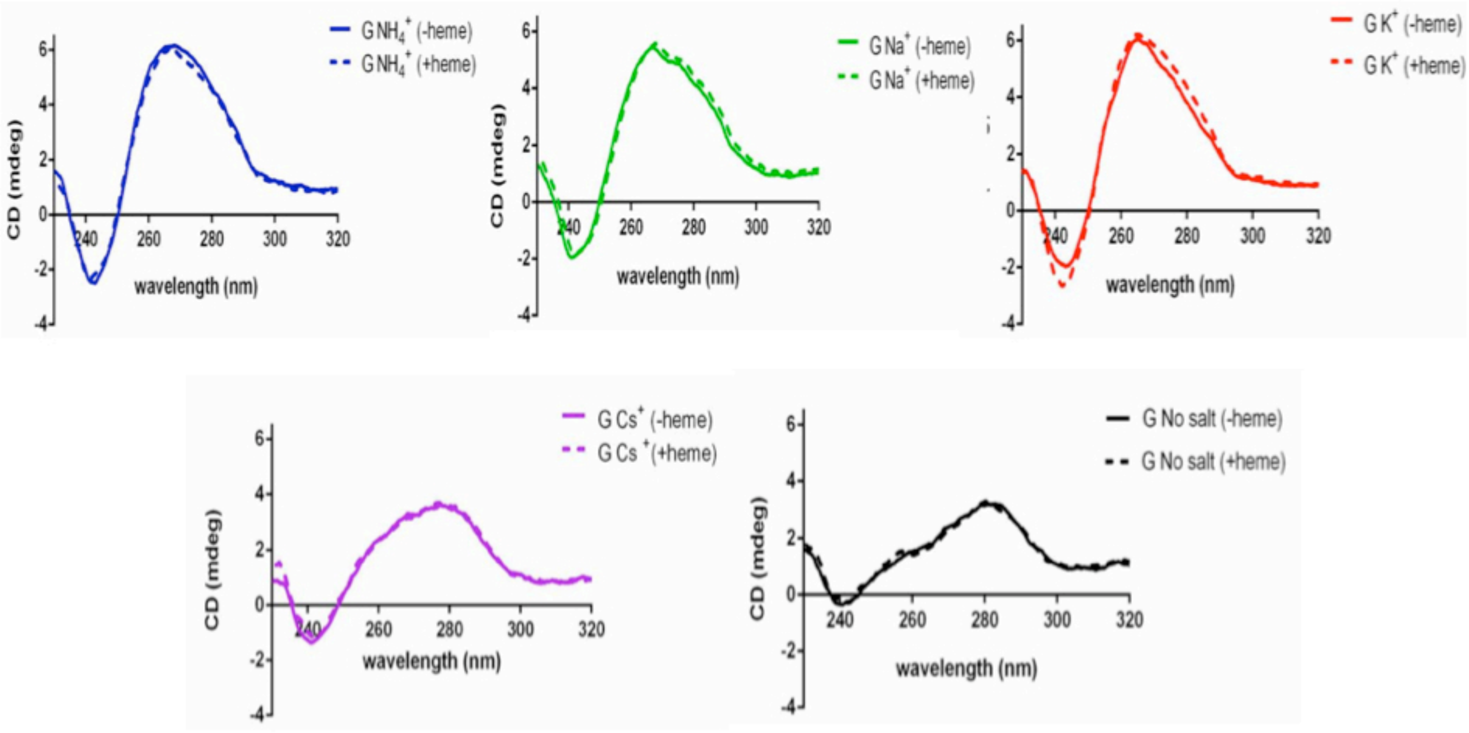

<u>Figure S3a</u>: Circular dichroism spectra, in the absence and presence of 0.5 μM heme, of G-quadruplexes formed by dT_8G_4T (G $NH_4^+/Na^+/K^+/Cs^+$) and of the single stranded DNA itself (G No salt).

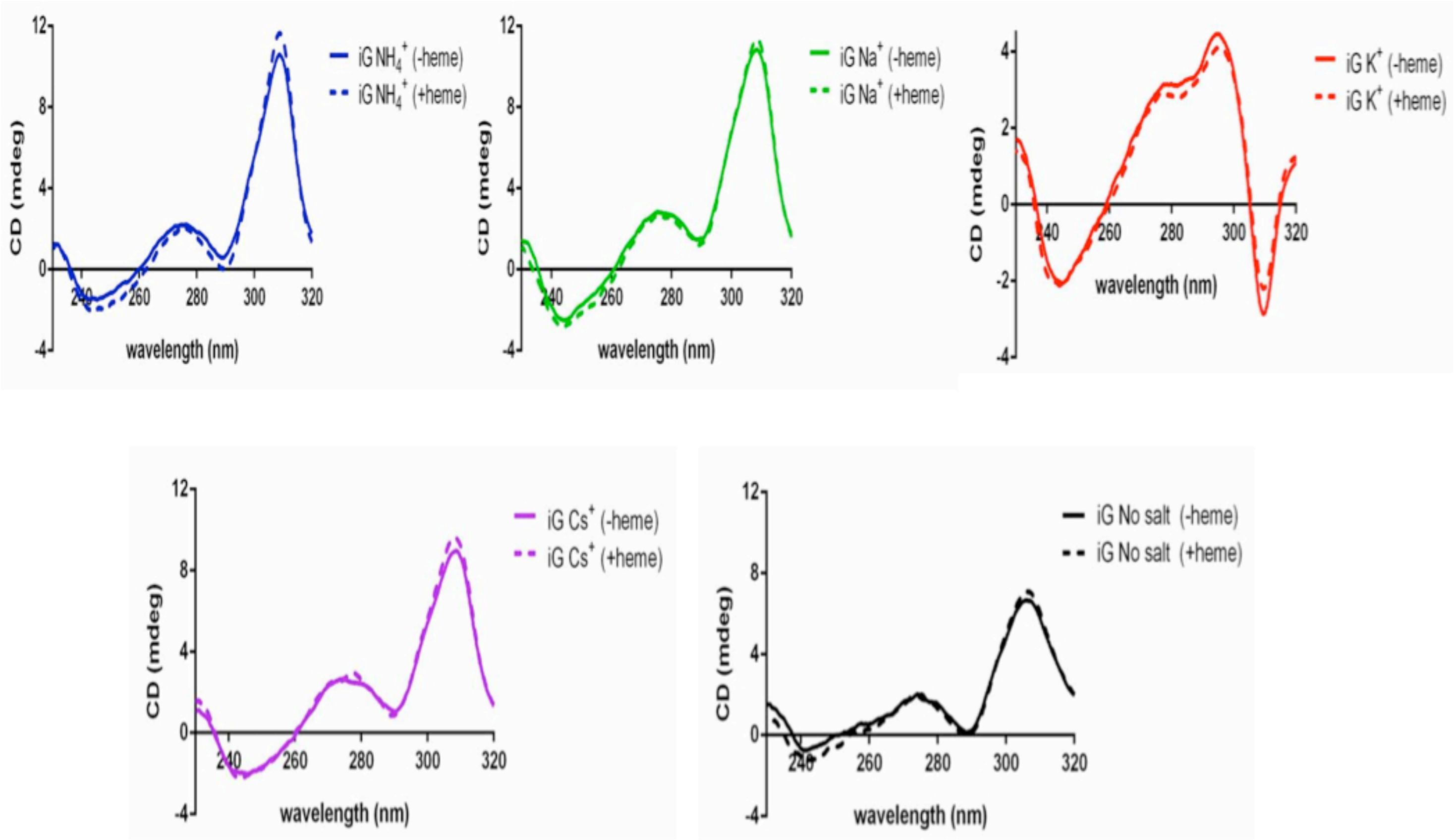

<u>Figure S3b</u>: Circular dichroism spectra, in the absence and presence of 0.5 μM heme, of iG-pentaplexes formed by dT_8iG_4T (G $NH_4^+/Na^+/Cs^+$), iG-quadruplex formed by dT_8iG_4T (G K^+), and of the single stranded DNA itself (G No salt).

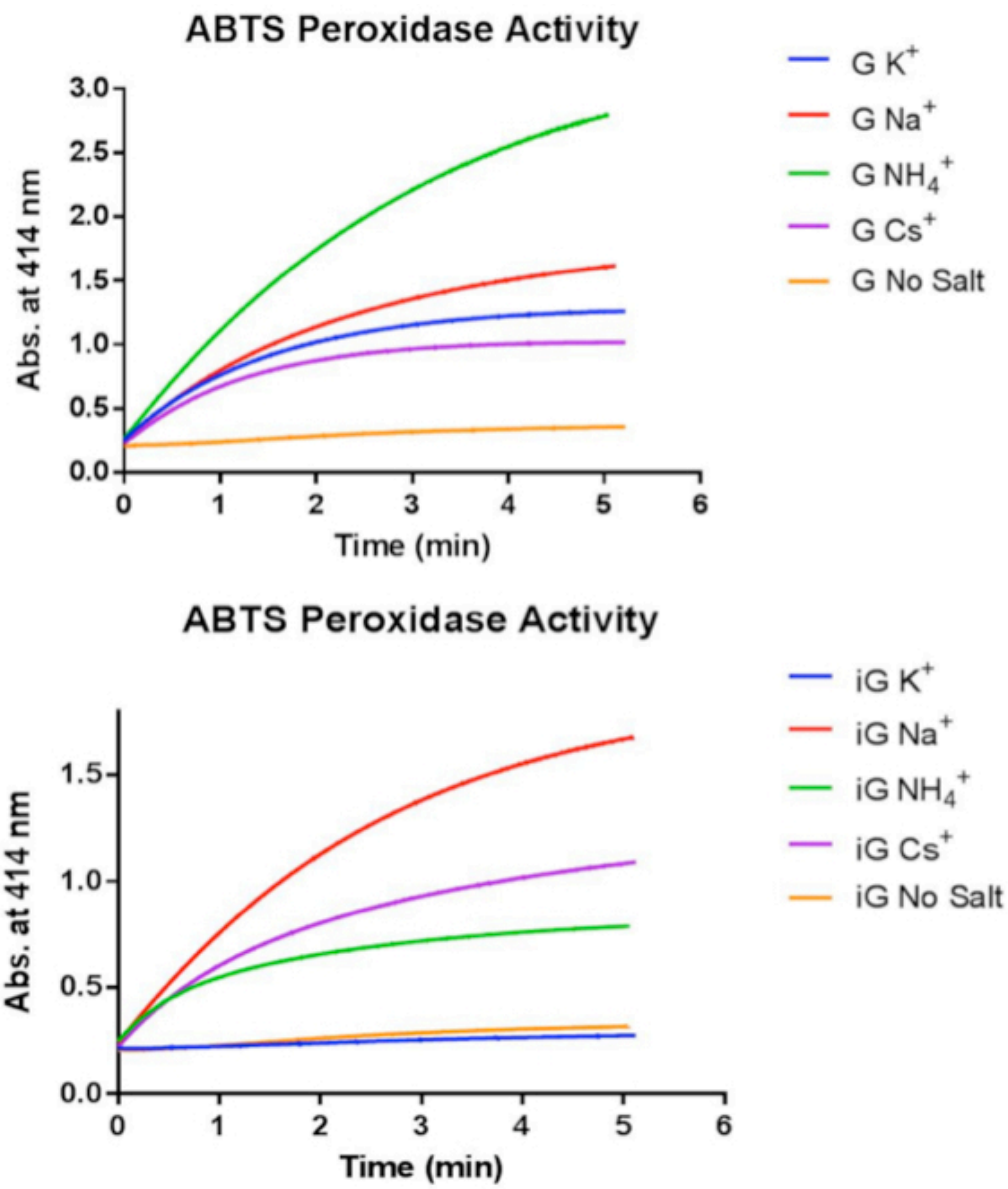

<u>Figure S4</u>: ABTS peroxidation as a functions of time. Reactions solutions contained heme (0.1 μM), in reaction buffer containing 20 mM of XCl (where X is Na, K, Cs, or NH_4). The "no salt" reactions were monitored in reaction buffer itself, with no XCl added. ABTS was at 5 mM and multi-stranded DNA at 20 μM, respectively. Reactions were initiated, at 25 °C, with the addition of 1 mM H_2O_2.

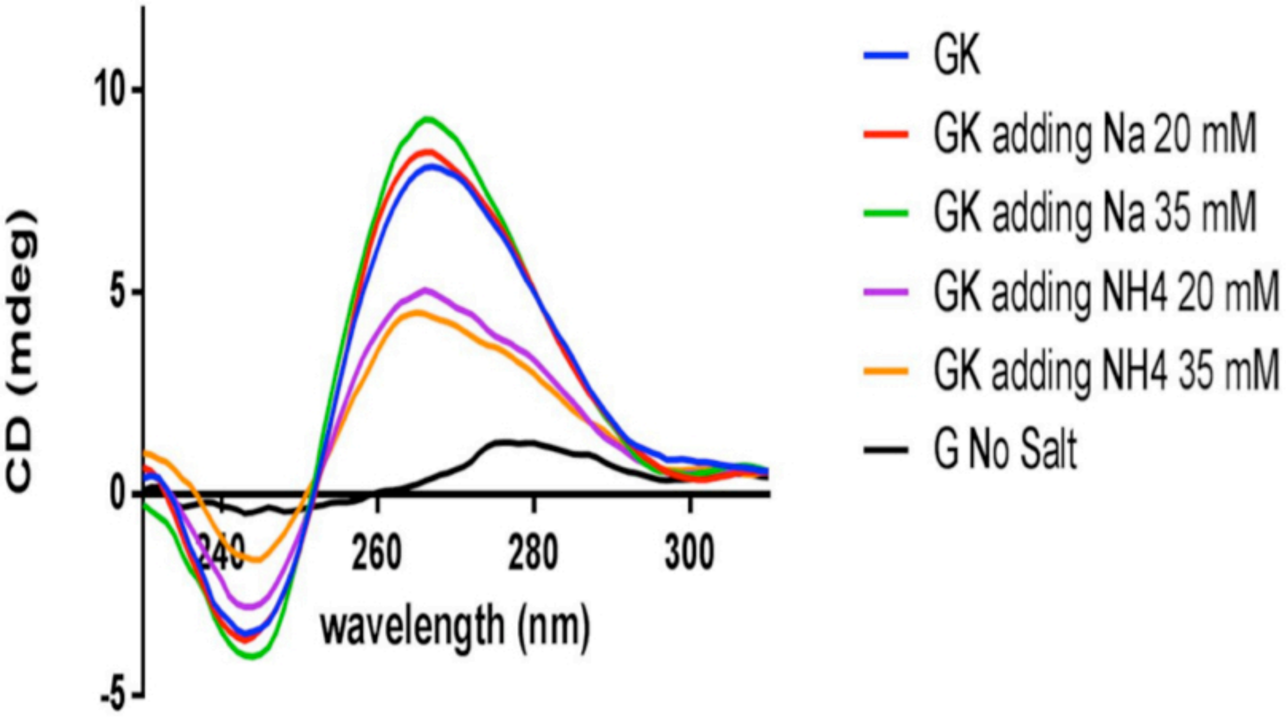

Figure S5a: Circular dichroism spectra of the G-quadruplex, $(T_8G_4T)_4$, formed in K^+-buffer ("GK"), and, following the addition of different concentrations of NaCl and NH_4Cl, as indicated.

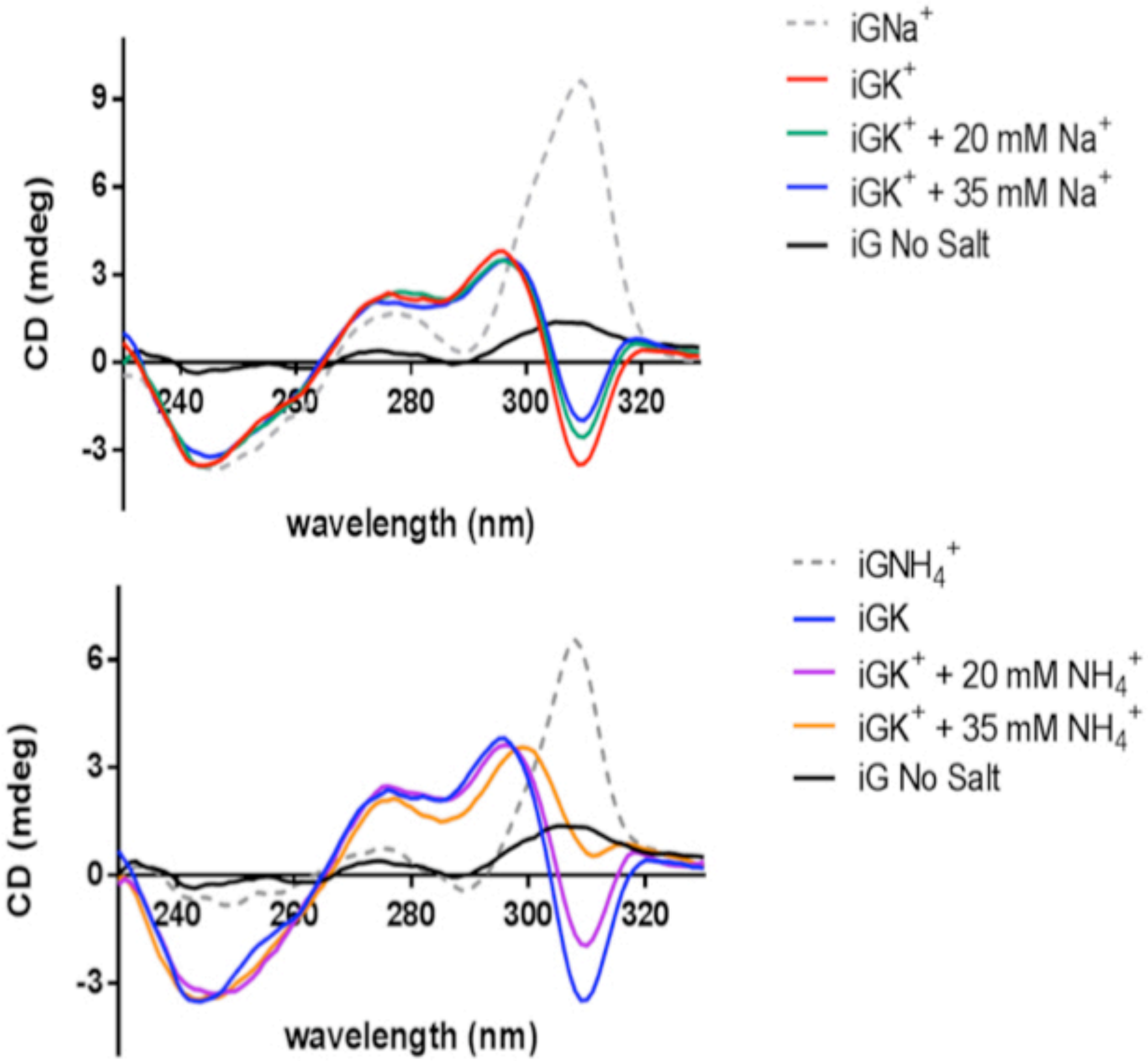

Figure S5b: Circular dichroism spectra of the iG-quadruplex, $(T_8iG_4T)_4$, formed in K^+-buffer ("iGK"), and, following the addition of different concentrations of NaCl and NH_4Cl, as indicated. "iGNa" indicates, for reference, the CD spectrum of the iG-pentaplex, $(T_8iG_4T)_5$, formed in Na^+-buffer.
